# *Helicobacter pylori* Antibodies and Iron Deficiency in Female Adolescents

**DOI:** 10.1371/journal.pone.0113059

**Published:** 2014-11-19

**Authors:** Göran Sandström, Stig Rödjer, Bertil Kaijser, Mats Börjesson

**Affiliations:** 1 Institute of Medicine, Dep. of Molecular and Clinical Medicine, Sahlgrenska University Hospital/Östra, Sahlgrenska Academy, University of Gothenburg, Sweden; 2 Dep. Of Medical Microbiology, Sahlgrenska University Hospital, Sahlgrenska Academy, University of Gothenburg, Sweden; 3 Swedish School of Sports and Health Sciences and Karolinska University Hospital, Stockholm, Sweden; National Cancer Center, Japan

## Abstract

**Objective:**

Iron deficiency (ID) is a common clinical problem worldwide, affecting primarily females. *Helicobacter pylori* (HP) infection has been shown to be associated with ID. The objective of this study was to define the prevalence of HP antibodies in female adolescents, and to find out if there was a correlation between HP infection and ID. The secondary aim was to study if regularly performed sporting activity, have any association to HP infection, in itself.

**Design:**

A controlled clinical trial.

**Setting:**

A senior high school in Gothenburg, Sweden.

**Subjects:**

All female athletes at a senior high school for top-level athletes were offered to take part, and 56 athletes took part in the study. The control group consisted of a random sample of age-matched non-athlete students of which 71 entered the study.

**Main outcome measures:**

Iron deficiency (ID) and iron deficiency anaemia (IDA) were defined by the use of levels of haemoglobin, serum iron, total iron-binding capacity, transferrin saturation, and serum ferritin, as previously described. HP IgG-antibodies were detected by ELISA.

**Results:**

18 of 127 (14%) adolescent females had antibodies against HP. Only 3% had IDA, while 50% had ID. In total, 66% of the HP positive females had ID compared to 48% of the negative females (p = 0.203). No correlation between sporting activity and HP infection was found. Regarding ethnicity, 11/28 of subjects from medium-high risk areas were HP-positive, compared to 7/99 coming from low-risk areas (p<0.001).

**Conclusion:**

The main finding of this study is that the prevalence of HP IgG antibodies was 14% in adolescent females. We could not find any difference regarding frequency of ID and IDA, between HP positive and negative individuals. Ethnicity is of great importance for the risk of HP infection, while sporting activity itself seems to have no association to HP-infection.

## Introduction

*Helicobacter pylori* (HP) is a very common infection, demonstrating a high prevalence worldwide. In 1982, HP was suggested as a causative agent of gastritis and peptic ulcer [1]. This finding, overnight, changed the view of the stomach as a sterile area. Today, HP is considered to be the most common cause of gastritis, which is a benign condition [2].

Although HP infects almost half the population in the world [3], the exact mechanism of how it infects the human stomach is still not clear. Many factors influence the incidence and prevalence, such as age, gender, genetic predisposition, ethnicity, education level and sanitation [4].

During the last few years, *Helicobacter pylori* infection has been identified as a reason for unexplained iron deficiency [5]–[7], and it is known that HP infection impairs the iron adsorption, and through blood loss, also increases the iron loss [8], [9].

Few studies of the prevalence of the HP infection in adolescent females in Sweden have been undertaken. One study from Sweden showed a total incidence of HP IgG antibodies of 17% in children between 10–12 years of age [10]. However when specifically looking at children born by Scandinavian parents, the prevalence was 2%. This in contrast to the group of children originating from high prevalence regions (The Middle East and Africa) where the prevalence was 55%. In another study 10% of the children aged 4–18 years were HP positive at some time [11].

Iron deficiency (ID) is a common problem both world-wide and in Sweden. We have shown that it is common - in both adolescent female athletes and non-athletes [12]. Adolescents are particularly susceptible to ID due to their high iron requirements [13].

We have earlier reported that female athletes, despite having a better dietary intake and less losses by menses, do not show a better iron status than non-athlete controls [12]. Which factors that potentially contribute to these findings are currently not known. However, as HP infection previously has been associated with ID [14] it is an obvious candidate for further study. One single study has previously considered the significance of physical activity in relation to HP infection [5], so data is clearly lacking.

Thus, the primary aim of this study was to estimate the prevalence of HP antibodies in a group of adolescent females and to find out if there was any correlation between HP antibodies and iron deficiency. The secondary aim was to study if sporting activity in itself is associated with HP infection.

## Methods

### Ethics

This study was approved by the Ethics Committee at Sahlgrenska Academy, at Gothenburg University (Ö-005-01). Informed written consent were obtained from next of kin, caretakers or guardians, for participants under 18 years of age, taking part in the study. Participants 18 years and older gave their written informed consent to participate. The methods used in this investigation were in accordance to the Helsinki Declaration of 1975 as revised in 1983.

### Subjects

We offered all female student athletes (n = 71), at a senior high school for top-level athletes to participate in the study. This particular school is accepting students from the city of Gothenburg as well as from the surrounding county. Regarding the athlete students, they are both locally recruited and also recruited from the whole country. An age-matched control group consisting of a random sample of 130 individuals, derived from the 500 non-athlete female students at the same school, were offered to take part in the study. A total of 149 healthy young female participants, 57 (81%) of invited athletes and 92 (71%) of invited controls, being 15 to 19 years old, took part in the initial investigation, as earlier has been described [12].

Three possible times for testing were offered to maximise inclusion. Exclusion criteria were pregnancy, on-going infection, and a history of haematological disease (except for iron deficiency anaemia).

We were not able to obtain enough serum from all participants to perform all analyses for this part of the study. Thus we finally included 56 individuals in the athlete group and 71 individuals in the non-athlete group. To address the issue with the 21 drop-outs in the control-group we compared these individuals with the 71 subjects taking part in the study using Mann Witney U-test. No significant differences were found between the two groups regarding the different anthropometric measures and laboratory values in table 1.

**Table 1:** Major laboratory findings in HP positive and negative subjects.

	HP* antibodies positive	95% CI	HP* antibodies negative	95% CI	p value
n	18 (14%)	8–22	109 (86%)	70–100	–
Athletes (n)	4 (7%)	6–57	52 (93%)	36–63	–
Non-athletes (n)	14 (20%)	42–100	57 (80%)	39–68	–
Length (m)	1.68 (±0.1)	1.6–1.8	1.67±(0.1)	1.6–1.8	0.167
Weight (kg)	60 (±14)	53–67	61 (±6)	59–62	0.107
BMI (kg/m^2^)	22.1 (±4.7)	20–24	21.9 (±2.2)	21–22	0.244
Hb (g/L)	134 (±7.6)	130–138	137 (±8.5)	135–139	0.093
MCV (fL)	88.0 (±6.2)	85–91	89.9 (±4.3)	89–91	0.261
S-Fe (µmol/L)	17.1 (±8.9)	13–22	15.6 (±6.8)	14–17	0.787
TIBC (µmol/L)	72.4 (±11.4)	67–78	76.0 (±11.5)	74–78	0.162
Transferrin saturation (%)	23.9 (±12.4)	18–30	20.9 (±9.2)	19–23	0.504
Ferritin (µg/L)	20.4 (±21.6)	10–31	20.5 (±14.5)	18–23	0.296

Results are expressed as mean and SD. Comparison between the groups were done by Mann-Whitney U-test. p<0.050 considered as statistically significant. CI  =  confidence interval.

* =  *Helicobacter pylori*.

Body mass index (BMI) was calculated from the weight (in kilograms) divided by the square of the height (in meters).

### Blood Samples

The blood samples were drawn at the school clinic at certain given times. All subjects were fasting from midnight, and venous blood samples were drawn between 10 and 12 AM, with the subjects in a semi-supine position. The subjects had not undertaken any training, the morning before the blood sampling. The concentrations of haemoglobin (Hb), erytrocyte-indicies and white cell count were determined the same day. Serum was kept frozen at −70°C, and analysis of serum iron (Fe), TIBC, and serum ferritin was performed according to standard laboratory procedures.

The concentration of haemoglobin was determined by the Technicon H2 method (Bayer Diagnostics, Tarrytown, New York). Serum iron was determined with a photometric method as a ferrozine complex on Hitachi 917 (Boehringer Mannheim, Indianapolis, Indiana). Total iron-binding capacity was calculated from measurements of serum transferrin with an immunochemical method on Hitachi 917. Transferrin saturation (TS) was the ratio of serum iron to TIBC expressed as a percentage. Serum ferritin was measured by an immunochemical method using a mouse monoclonal antiferritin antibody and determined by alkaline phosphate conjugation according to AxSYM system (Abbot Laboratories, Abbot Park, Illinois).

*Helicobacter pylori* IgG antibodies was analysed using the enzyme linked immune-sorbent assay, ELISA, produced by EUROIMMUNE, Lübeck, Germany (www.euroimmune.de). The result was reported as relative units (RU). More than >22 RU/mL was considered as positive [15].

### Definitions

Anaemia was defined as a haemoglobin value <120 g/L as according to the WHO definition [16].

Iron deficiency was defined as a serum ferritin <16 µg/L according to the definition previously used [17].

### Statistics

We used commercially available statistical software (IBM SPSS Statistics for Windows, Version 22.0. Armonk, NY: IBM Corp) to perform the statistical analysis. Descriptive statistics are presented as mean ± SD and CI. For comparison of means between the groups we used Mann-Whitney U-test. All tests were two-sided. p<0.050 was considered statistically significant. Comparison between groups regarding variables that are dichotomous was performed with Fisher's exact test, and the following variables were compared: HP antibodies pos/neg and iron deficiency or not iron deficiency; HP antibodies pos/neg and athletes or non-athletes and finally HP antibodies pos/neg and origin from low risk area or high/intermediate risk area.

## Results

As shown in Table 1, a total of 18 of 127 adolescent females were positive for HP IgG (14%). One female in the HP positive group and three females in the HP negative group had anaemia according to the WHO definition [16], showing a haemoglobin <120 g/L.

The mean haemoglobin value in the HP positive group was 133.6 g/L and in the HP negative group 137.0 g/L, which is not statistically significant (p = 0.093).

In the whole group, 64 females (50%) had ID. Of those 64, 12 were HP positive and 52 HP negative. In the group of non-iron deficient females, (n = 63), we found 6 with positive HP serology. There was no statistical significant difference in HP positive subjects comparing iron deficient subjects with non-iron deficient subjects, (p = 0.203), Table 2.

**Table 2:** The distribution of the study population related to *Helicobacter pylori* (HP) antibodies and iron deficiency.

	HP antibodies positive	HP antibodies negative	
**Iron deficiency**	12	52	64
**No iron deficiency**	6	57	63
	18	109	127

Difference not significant, p = 0.203 (Fisher's exact test).

We found none with IDA and one with ID among the four HP positive athletes. In the group of HP positive non-athletes (n = 14) there was one with IDA and eleven with ID.

There were no differences in serum iron (p = 0.787), TIBC (p = 0.162), transferrin saturation (p = 0.504), or ferritin (p = 0.296) between HP positive and negative individuals (Table 1).

No correlation between sporting activity and HP infection was found (Table 3). In the group of seropositive females in our study, as many as 11 (61%), originate from areas classified as being intermediate or high-risk areas (Table 4).

**Table 3:** The distribution of the study population related to *Helicobacter pylori* (HP) antibodies and if the subjects are athletes or non-athletes.

	HP antibodies positive	HP antibodies negative	
**Athletes**	4	52	56
**Non-athletes**	14	57	71
	18	109	127

Difference not significant, p = 0.071 (Fisher's exact test).

**Table 4:** The distribution of the study population related to *Helicobacter pylori* (HP) antibodies and origin from low-, intermediate- or high-risk areas.

	HP antibodies positive	HP antibodies negative	
**Low risk areas**	7	92	99
**Intermediate and high risk areas**	11	17	28
	18	109	127

Difference statistically significant, p<0.001 (Fisher's exact test).

There was no significant difference between HP positive and HP negative subjects regarding BMI and weight.

## Discussion

The main finding of this study, is that 14% of adolescent females were shown to be HP positive. Out of the HP positive individuals, 66% were iron deficient compared to 48% of the HP negative individuals. This difference was not statistically significant. We could not demonstrate a statistically significant difference between athletes and non-athletes regarding the occurrence of HP-positivity, with a p-value of 0.071 (Table 3).

There were no significant differences in laboratory findings between HP positive and HP negative individuals, although it has previously been reported that HP infected individuals may express higher values of s-ferritin [7].

Interestingly, in our study, the prevalence of IgG antibodies for HP in this group of young females seems higher than previously reported [10], [11]. The prevalence of HP positivity is 14% for the whole group with 7% of the athletes and 17% of the non-athletes being IgG positive. Previous prevalence figures in Sweden have been lower and not many reports have previously pinpointed this age group. In a study by Thjodleifsson and collaborators from Iceland, Sweden and Estonia, the HP-prevalence in participants under age of 30, was only 3.8% [18].

*Helicobacter pylori* is reported to be acquired in early childhood [19], and the risk of infection seems to be linked to the family background. In one study three different levels of risk areas have been proposed; low risk (origin in Scandinavia, Western Europe, and the United States); medium risk (Eastern Europe, South America, Asia), and high risk (Middle East, Africa) [10]. Tindberg et al in 2001 reported a HP-prevalence of 2% among children with Scandinavian parents and 55% among children originating from high risk areas (Middle East and Africa). The high prevalence of HP antibodies in our study subjects is most likely related to the geographical origin of the individual, with 61% in the intermediate/high risk group.

ID is still a very common condition worldwide and most commonly affects females. It is also a well-known condition in female athletes, with a prevalence figure as high as 55% of female athletes, being reported [20]. Normally, it is not difficult to establish a clear diagnosis of ID, which in most subjects is explained by an inadequate dietary intake or losses by menses, or a combination of both. However in some cases other explanations for the ID must be excluded, as e.g. gastrointestinal disorders like celiac disease and gastrointestinal bleeding.

During the last decade HP infection has been discussed as a possible cause of ID and IDA [8], [9], [21]. Muhsen et al have reported that HP infection can be regarded as a potential risk factor for iron deficiency. The mechanism is not fully understood, but it seems to involve several pathways, including gastrointestinal blood loss and decrease in iron absorption [9]. It has also been proposed that the HP bacteria consume iron for growth and therefore compete with the host [22].

A reduced effect of iron supplementation was seen in one study [9] and in another study eradication of HP improved the haemoglobin value in patients suffering from IDA [14].

In individuals with ID and not responding to iron supplementation, the compliance to iron supplementation must first be evaluated. If the compliance is found to be sufficient other reasons must be evaluated. As a result of the present study, we propose that a *H. pylori* test should be included in the gastrointestinal evaluation, especially in individuals originating from intermediate and high-risk areas. If IgG positivity is found the diagnosis of active infection can be verified by urea breath test for example. If the diagnosis of HP is in ID-patients, eradication treatment can be instituted.

## Limitations

We could not demonstrate a statistically significant difference in the occurrence of ID between HP positive and HP negative individuals. The association between being HP positive, and iron deficient, thus needs to be addressed in a larger trial.

A potential confounding factor in this study is may be that the control group was recruited locally, whereas the athlete group originated from all over Sweden. This may have contributed to the higher proportion of subjects in the control group originating from intermediate and high risk areas regarding HP infection.

## Conclusions

*Helicobacter pylori* antibodies are relatively common in adolescent females (1 of 7) and ID is very common, affecting one of two. HP is more common in adolescents with parents originating from high-risk areas. Regular intensive sporting activity does not seem to be a risk factor for *Helicobacter pylori* infection per se, but must be considered in the context of increased iron demands related to adolescence. In a young adolescent with ID, not responding to oral iron supplementation, the presence of HP infection should be considered, especially if the youngster originates from high-risk areas.
